# Comparison of the Efficacy of Ultrasound-Guided Suprascapular Nerve Block Versus Intra-articular Platelet-Rich Plasma in Periarthritis Shoulder Pain: A Randomized Controlled Trial

**DOI:** 10.7759/cureus.94415

**Published:** 2025-10-12

**Authors:** Vinay Kanaujia, Dhirendra Kumar, Javed Ahmad, Shipra Verma, Sanjai Singh, Chandan Sharma

**Affiliations:** 1 Physical Medicine and Rehabilitation, Uttar Pradesh University of Medical Sciences, Etawah, IND; 2 Anaesthesiology, Uttar Pradesh University of Medical Sciences, Etawah, IND

**Keywords:** frozen shoulder, periarthritis shoulder, platelet-rich plasma, shoulder pain and disability index (spadi), suprascapular nerve block

## Abstract

Purpose: The study aimed to compare the effect of ultrasound-guided suprascapular nerve block (SSNB) to intra-articular platelet-rich plasma (PRP) in the management of periarthritis shoulder (PA shoulder).

Methods: This study was a single-center randomized, interventional clinical trial conducted in the Department of Physical Medicine and Rehabilitation of Uttar Pradesh University of Medical Sciences (UPUMS), a tertiary care teaching hospital in Saifai, Etawah, over a period of 12 months from April 2024 to April 2025. Sixty patients were randomly divided into two groups (30 each). Group A received 3 ml of PRP under ultrasound guidance, and Group B underwent ultrasound-guided SSNB using 10 ml of 0.5% bupivacaine. Outcomes were assessed at the baseline and at the second, sixth, and 12th weeks post-intervention using the Shoulder Pain and Disability Index (SPADI) scale, Visual Analogue Scale (VAS), and range of motion (ROM).

Results: Both groups showed significant improvement across all measures over time. However, on intergroup comparison, the total SPADI score was insignificant at two weeks, but it became significant at six and 12 weeks in favour of the PRP group. The VAS score also favoured the PRP group throughout the study period. In the ROM, flexion and abduction were significantly improved in the PRP group, while external rotation showed no significant difference at the second week but became significant at six and 12 weeks.

Conclusions: Both USG-guided IA-PRP and SSNB are effective in PA shoulder pain management, but IA-PRP yields superior outcomes in the short to medium term.

## Introduction

According to Codman, “frozen shoulder” is a condition that is “difficult to define, difficult to treat, and difficult to explain from the point of view of pathology” [1]. The American Academy of Orthopedic Surgeons defines periarthritis shoulder (PA shoulder) as a condition of uncertain etiology characterized by significant restriction of both active and passive range of motion (ROM) that occurs in the absence of a known intrinsic shoulder disorder [2]. This condition, also referred to as “adhesive capsulitis” and “frozen shoulder,” has a prevalence of 2-5% in the general population. It is two to four times more common in women than in men and affects middle-aged and elderly people [2].

PA shoulder is characterized by painful, restricted shoulder ROM in patients with normal radiographs. The treatment includes nonsteroidal anti-inflammatory drugs (NSAIDs), electrotherapy, exercise regimens, intraarticular steroid injections, suprascapular nerve blocks (SSNBs), manipulation under anesthesia, hydrodilatation, arthroscopic capsular release, and recent modalities such as platelet-rich plasma (PRP) [3].

Of these, SSNB is a safe and effective treatment for chronic shoulder pain in conditions such as PA, irreparable rotator cuff tear, calcific tendinitis, and stroke sequelae. The suprascapular nerve originates from the upper trunk of the brachial plexus and travels to the shoulder, supplying sensation to the glenohumeral and acromioclavicular joints. Furthermore, this nerve provides motor innervation to the supraspinatus and infraspinatus muscles. The primary mechanism involves blocking the sensory fibers of the suprascapular nerve, which carry pain signals from the shoulder joint. By reducing pain, an SSNB can improve shoulder ROM and facilitate rehabilitation exercises [4]. PRP is a component of plasma that contains a high concentration of platelets and growth factors. PRP is obtained after centrifugation of the patient’s own blood. Being rich in growth factors, it is believed to increase vascularity and improve the healing process. Considering these properties, intraarticular PRP (IA-PRP) injections might prove beneficial for patients with PA shoulder. Therefore, its efficacy must be examined and compared with that of other available treatments [5].

Despite the availability of the above treatment options for years, high-quality evidence comparing the neural blockade technique with the regenerative intra-articular intervention for managing PA shoulder is limited. Trials have so far been conducted only on the broader shoulder pain syndrome or in isolation. This study, in which ultrasonography (USG)-guided IA-PRP and SSNB interventions, two highly utilized modalities with distinct mechanisms of action, were employed for the management of PA shoulder, bridges the research gap.

## Materials and methods

This study was a single-center randomized, interventional clinical trial conducted in the Department of Physical Medicine and Rehabilitation of Uttar Pradesh University of Medical Sciences (UPUMS), a tertiary care teaching hospital in Saifai, Etawah, over a period of 12 months from April 2024 to April 2025. A study by Kothari et al. [6] observed that the visual analog scale (VAS) values at 12 weeks in PRP and steroid therapies were 1.9 ± 1.8 and 3.4 ± 2.2, respectively. Taking these values as a reference, the minimum required sample size with 80% power of the study and 5% level of significance was 29 patients in each study group. To reduce the margin of error, the total sample size taken was 30 (30 patients per group). The following formula was used for sample size calculation:

\[ N = 2 \cdot (s_p^2) \cdot \frac{\big[ Z_{1-\alpha/2} + Z_{1-\beta} \big]^2} {(\Delta)^2} \]

where Z(1 − α/2) is the standardized normal deviation (two-tailed); at α = 0.05 and 95% CL, it is 1.96. Z (1 − β), which depends upon power; for 80%, it is 0.84. The mean difference is the difference in mean values of the two groups.

**Figure 1:**
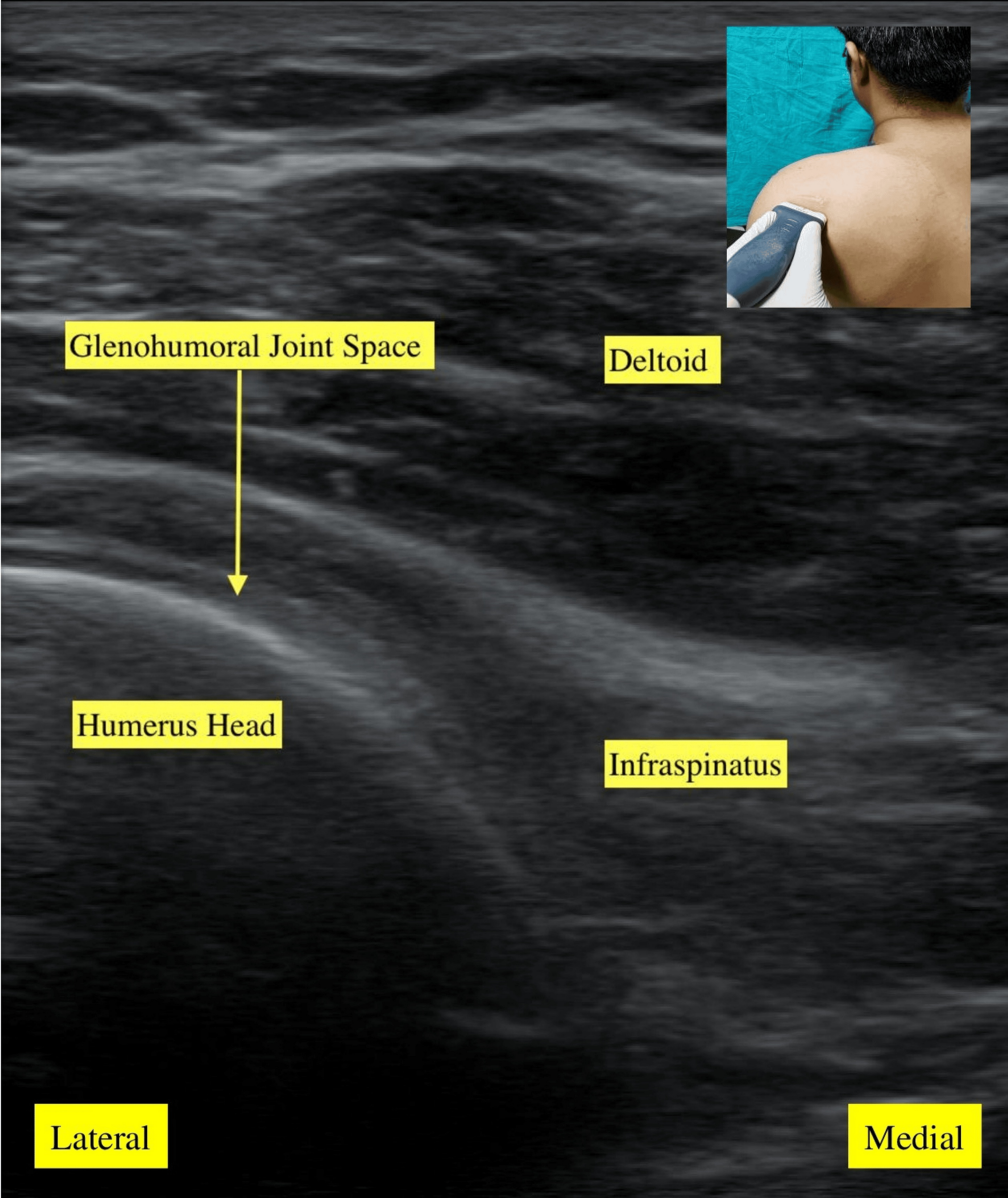
Shoulder joint sonoanatomy

**Figure 2:**
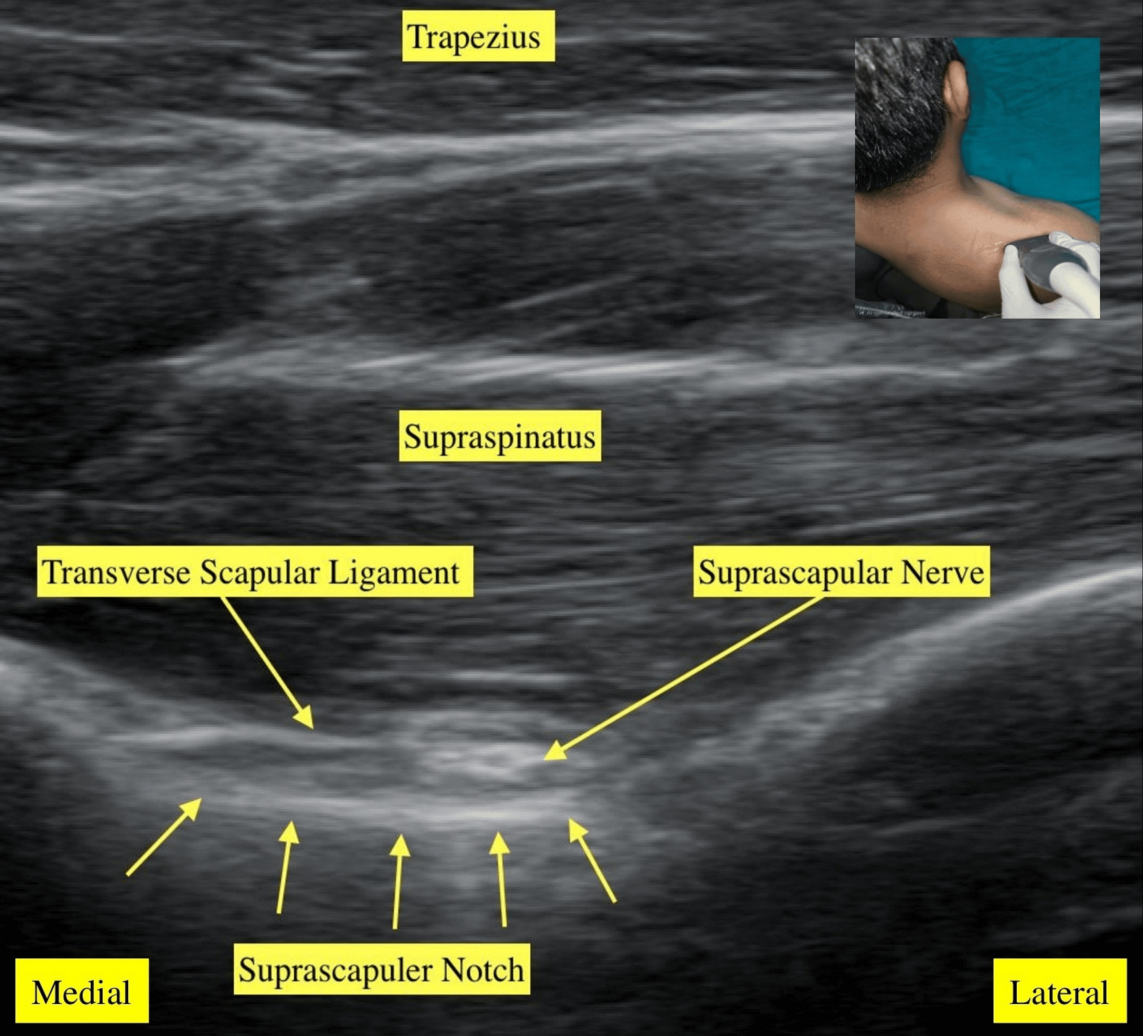
Suprascapular nerve sonoanatomy

This study was initiated after obtaining approval from the Institutional Ethics Committee of Uttar Pradesh University of Medical Sciences (UPUMS), Saifai, Etawah (approval no. 168/2023-24), and was subsequently registered in the Clinical Trial Registry-India (CTRI/2024/04/065537), dated 10/04/2024. All patients visiting the outpatient department with complaints of shoulder pain and restricted ROM were thoroughly examined and screened for PA shoulder. Patients clinically diagnosed with PA shoulder were enrolled in the study after obtaining written informed consent and applying the inclusion and exclusion criteria. The inclusion criteria for the patients were an age of >18 years, a clinical diagnosis of PA of the shoulder, a symptom duration of >4 weeks, and normal anteroposterior radiographs of the glenohumeral joint. Patients with a history of shoulder trauma, dislocation, or fractures in the shoulder area; a history of injection in the involved shoulder during the preceding six months; bleeding disorders; uncontrolled diabetes mellitus; active infection at the site of injection; a history of allergy to local anesthetics; use of anticoagulant drugs in the past seven days; surgery in the affected shoulder in the past one year; and those who were pregnant or breastfeeding were excluded from the study. Baseline evaluations were conducted and recorded for all selected patients in a prestructured proforma before the intervention. The participants were randomized into two groups using the RANDBETWEEN feature of Microsoft Excel (Microsoft Corp., USA) and allocated into two parallel groups with an allocation ratio of 1:1. In this study, the interventionist and the assessor were different to maintain the blinding. Under aseptic preparations, Group A underwent USG-guided single-shot IA-PRP application, and Group B underwent USG-guided SSNB. The sonographic anatomies of the shoulder joint and suprascapular nerve were identified using the linear probe (3-17 MHz) of the USG machine (BPL E-CUBE i7 model) and depicted in Figure 1 and Figure 2, respectively.

**Figure 3:**
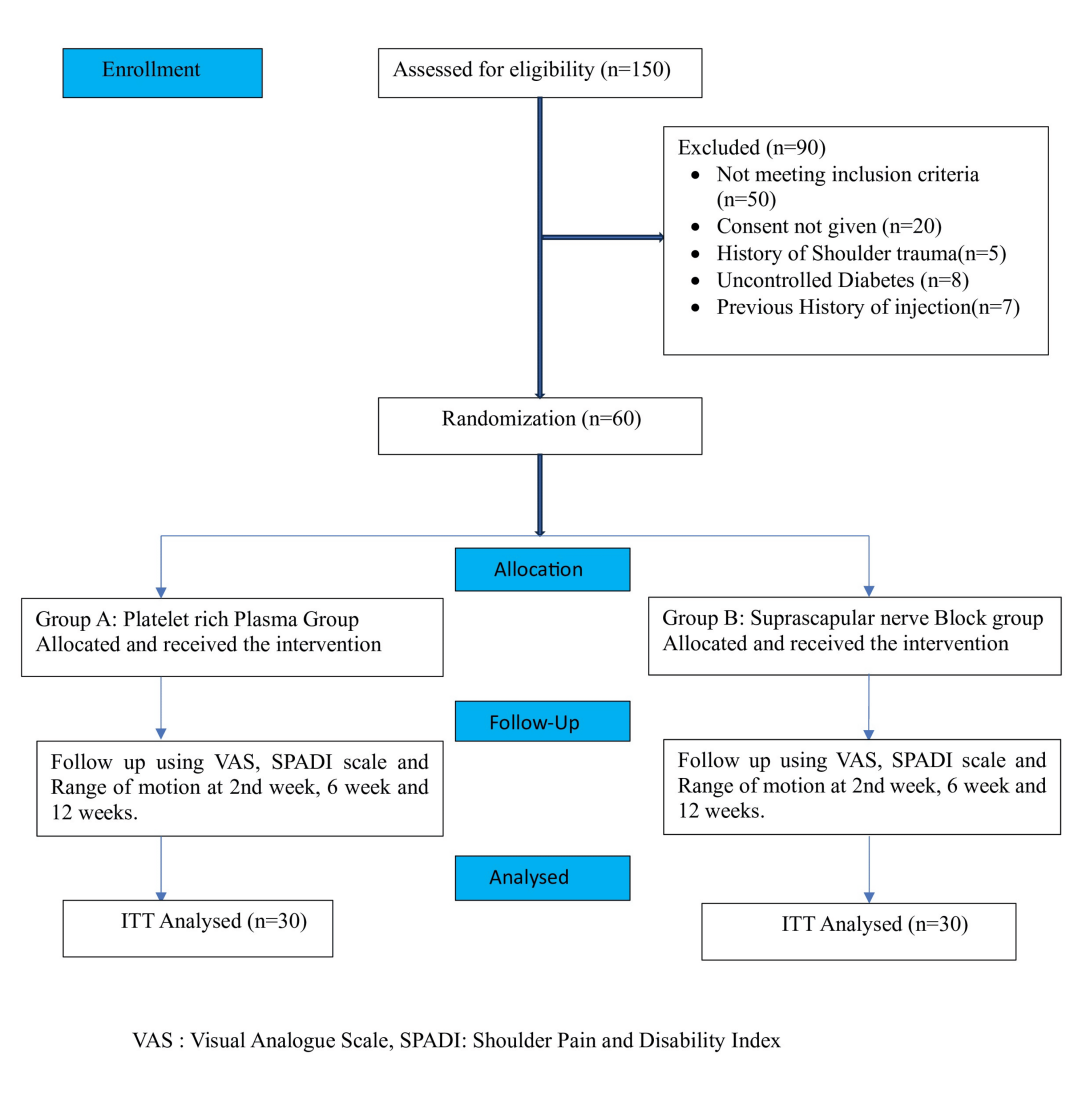
Consort flow chart

PRP was prepared using the HERMLE-Z206A Compact Centrifuge with the double centrifugation technique (500 g for 10 minutes as soft spin, and subsequently, 2,000 g for 10 minutes as hard spin) at an optimum temperature (20°C-24°C). All PRP preparation steps were performed using strict aseptic techniques. The platelet counts of PRP were compared with the baseline count. The preparation was injected into the affected shoulder only when the count was more than three times the baseline. For activation, 0.1 mL of 10% CaCl2 was used prior to the injection. The CONSORT flow chart of the study is presented in Figure 3.

NSAIDs or other analgesic medications were not allowed during the study period. Tablet acetaminophen (maximum dose of 2 g/day) was advised if the pain was intolerable, and it was recorded.

Group A

In Group A, 30 mL of blood was collected from the patients. After centrifugation, 3 mL of PRP was acquired. The patient was positioned seated with the shoulder internally rotated and the hand resting on the contralateral shoulder to optimize exposure of the posterior joint space. The ultrasound probe was placed in the long axis of the infraspinatus tendon, parallel to and just inferior to the scapular spine, to visualize the posterior glenohumeral joint. An in-plane technique was used to administer the PRP injection.

Group B

On the affected side of the shoulder, the USG transducer was positioned parallel to the scapular spine to visualize the bony landmark. Sliding the transducer cephalad revealed the suprascapular fossa. While imaging the supraspinatus muscle and the underlying fossa, the transducer was gradually shifted laterally, maintaining its transverse orientation, to identify the suprascapular notch. The suprascapular nerve was visualized as a round, hyperechoic structure at a depth of approximately 4 cm, located beneath the transverse scapular ligament within the notch. Under adequate aseptic precautions, the suprascapular nerve was infiltrated with 10 mL of 0.5% bupivacaine using a 21-gauge needle and a 10 mL syringe.

A uniform home-based exercise program was taught to all the patients of both groups. The patients were clinically assessed using a VAS, in which the pain was scored on a scale of 0-10 (0 = no pain, 10 = most severe pain) [7], and the shoulder pain and disability index (SPADI) scale, with appropriate permission [8]. It is a self-administered questionnaire that comprises two dimensions, one for pain and the other for functional activities. The pain dimension includes five questions on the severity of an individual’s pain. Functional activities were evaluated with eight questions designed to measure the degree of difficulty experienced by the individuals during various activities of daily living requiring upper-extremity use [8]. Active ROM measurement was performed using a 360-degree goniometer (on baseline day 0, week 2, week 6, and week 12).

Statistical analysis

Categorical variables were presented in the form of numbers and percentages (%). On the contrary, normally distributed quantitative data were presented as means ± SD, and data with a non-normal distribution as medians with 25th and 75th percentiles (interquartile range). The normality of the data was checked using the Shapiro-Wilk test. In cases where the data were not normally distributed, nonparametric tests were used. Quantitative variables that were not normally distributed were compared and analyzed using the Mann-Whitney test, and variables that were quantitative and normally distributed were analyzed using an independent samples t-test. Furthermore, the Wilcoxon signed-rank test was used for comparison across follow-ups. Qualitative variables were compared and analyzed using the chi-square test. The data entry was performed using Microsoft Excel, and the final analysis was conducted with the IBM SPSS Statistics, version 25.0 (IBM Corp., Armonk, NY). A p-value of <0.05 was considered to indicate statistical significance.

## Results

A total of 60 patients participated in the study, with a mean age of 54.65 ± 5.3 years and a female preponderance in the ratio of 1:1.5 (M:F). The right side was predominantly involved in 71.67% of the patient population. The detailed baseline comparison of the patient characteristics is furnished in Table 1.

**Table 1:** Comparison of baseline characteristics between Group A and Group B ‡ Independent t test, * Mann-Whitney test, † Chi-square test SPADI: Shoulder Pain and Disability Index, VAS: Visual Analogue Scale

Baseline characteristics	SSNB (n = 30)	PRP (n = 30)	Total	p-value	Test value
Age (years)	53.47 ± 5.04	55.83 ± 5.37	54.65 ± 5.3	0.084^‡^	t-test = 1.76
Gender					
Male	12 (40%)	12 (40%)	24 (40%)	1^†^	Chi-square test = 0
Female	18 (60%)	18 (60%)	36 (60%)		
Affected side					
Left	9 (30%)	8 (26.67%)	17 (28.33%)	0.774^†^	Chi-square test = 0.082
Right	21 (70%)	22 (73.33%)	43 (71.67%)		
Pain score	42 (40-44)	42 (40-44)	42 (40-44)	0.828^*^	Mann-Whitney test = 435.5
Disability score	71.5 (70-73.5)	72 (70-74)	72 (70-74)	0.756^*^	Mann-Whitney test = 429.5
Total SPADI score	112 (110-116.75)	112 (110-116)	112 (110-116)	0.947^*^	Mann-Whitney test = 445.5
VAS score	8 (8-8)	8 (8-8)	8 (8-8)	0.657^*^	Mann-Whitney test = 425
Flexion (°)	89 (85-90)	87.5 (80-90)	89 (80-90)	0.487^*^	Mann-Whitney test = 404.5
Abduction (°)	82 (80-88.75)	85 (80-90)	85 (80-90)	0.128^*^	Mann-Whitney test = 350.5
External rotation (°)	15 (15-20)	20 (15-20)	20 (15-20)	0.213^*^	Mann-Whitney test = 373

In Group A, significant improvements were observed in all parameters at two, six, and 12 weeks compared with the baseline (p-value < 0.0001 for all comparisons). The total SPADI score improved significantly at two, six, and 12 weeks (p-value < 0.0001). The VAS score decreased at two, six, and 12 weeks (p-value < 0.0001). Moreover, significant improvements were observed in flexion, abduction, and external rotation ROM, which increased at two, six, and 12 weeks (p-value < 0.0001) (see Table 2).

**Table 2:** Comparison of parameters between baseline and at follow-up in Group A ¶ Wilcoxon signed-rank test SPADI: Shoulder Pain and Disability Index, VAS: Visual Analogue Scale

Parameters	At baseline (n = 30)	At two weeks (n = 30)	At six weeks (n = 30)	At 12 weeks (n = 30)
Pain score	42 (40-44)	30 (23.25-33.5)	20 (16-22)	14 (11-15.5)
Intragroup p-value	-	<0.0001^¶^	<0.0001^¶^	<0.0001^¶^
Z value		-4.822	-4.804	-4.805
Disability score	71.5 (70-73.5)	60 (58-61.5)	39 (36-42)	23 (20-28)
Intragroup p-value	-	<0.0001^¶^	<0.0001^¶^	<0.0001^¶^
Z value		-4.85	-4.797	-4.801
Total SPADI score	112 (110-116.75)	89 (82.25-93.5)	58 (54.25-62)	38 (32-42)
Intragroup p-value	-	<0.0001^¶^	<0.0001^¶^	<0.0001^¶^
Z value		-4.801	-4.796	-4.795
VAS score	8 (8-8)	5 (4-5)	4 (3-4)	2 (2-3)
Intragroup p-value	-	<0.0001^¶^	<0.0001^¶^	<0.0001^¶^
Z value		-4.902	-4.926	-4.893
Flexion (°)	89 (85-90)	110 (100-120)	125 (120-135)	140 (135-150)
Intragroup p-value	-	<0.0001^¶^	<0.0001^¶^	<0.0001^¶^
Z value		-4.832	-4.798	-4.8
Abduction (°)	82 (80-88.75)	100 (95-100)	120 (115-120)	140 (140-145)
Intragroup p-value	-	<0.0001^¶^	<0.0001^¶^	<0.0001^¶^
Z value		-4.808	-4.8	-4.809
External rotation (°)	15 (15-20)	25 (25-30)	45 (45-45)	50 (45-50)
Intragroup p-value	-	<0.0001^¶^	<0.0001^¶^	<0.0001^¶^
Z value		-4.659	-4.852	-4.830

In Group B, significant improvements were observed in all parameters at two, six, and 12 weeks (p-value < 0.0001 for all comparisons) from the baseline. The total SPADI score improved significantly from the baseline at two, six, and 12 weeks (p-value < 0.0001). The VAS score decreased at two, six, and 12 weeks (p-value < 0.0001). Flexion, abduction, and external rotation ROM increased from the baseline at two, six, and 12 weeks (p-value < 0.0001) (see Table 3).

**Table 3:** Comparison of parameters between baseline and at follow-up in Group B ¶ Wilcoxon signed-rank test SPADI: Shoulder Pain and Disability Index, VAS: Visual Analogue Scale

Parameters	At baseline (n = 30)	At two weeks (n = 30)	At six weeks (n = 30)	At 12 weeks (n = 30)
Pain score	42 (40-44)	28 (26-30)	22 (20.25-24)	16 (16-17.5)
Intragroup p-value	-	<0.0001^¶^	<0.0001^¶^	<0.0001^¶^
Z value		-4.801	-4.8	-4.805
Disability score	72 (70-74)	62 (60-64)	47 (42-48)	28 (26-32)
Intragroup p-value	-	<0.0001^¶^	<0.0001^¶^	<0.0001^¶^
Z value		-4.809	-4.809	-4.791
Total SPADI score	112 (110-116)	90 (88-92.75)	66 (62.5-70)	46 (40-50)
Intragroup p-value	-	<0.0001^¶^	<0.0001^¶^	<0.0001^¶^
Z value		-4.792	-4.789	-4.792
VAS score	8 (8-8)	6.5 (6-7)	5 (5-5)	3 (3-3)
Intragroup p-value	-	<0.0001^¶^	<0.0001^¶^	<0.0001^¶^
Z value		-4.939	-4.939	-4.965
Flexion (°)	87.5 (80-90)	100 (100-110)	120 (110-120)	130 (130-137.5)
Intragroup p-value	-	<0.0001^¶^	<0.0001^¶^	<0.0001^¶^
Z value		-4.899	-4.848	-4.831
Abduction (°)	85 (80-90)	90 (90-100)	110 (100-110)	122.5 (120-130)
Intragroup p-value	-	<0.0001^¶^	<0.0001^¶^	<0.0001^¶^
Z value		-4.75	-4.847	-4.806
External rotation (°)	20 (15-20)	25 (25-30)	35 (35-40)	40 (40-45)
Intragroup p-value	-	<0.0001^¶^	<0.0001^¶^	<0.0001^¶^
Z value		-4.835	-4.845	-4.877

**Figure 4:**
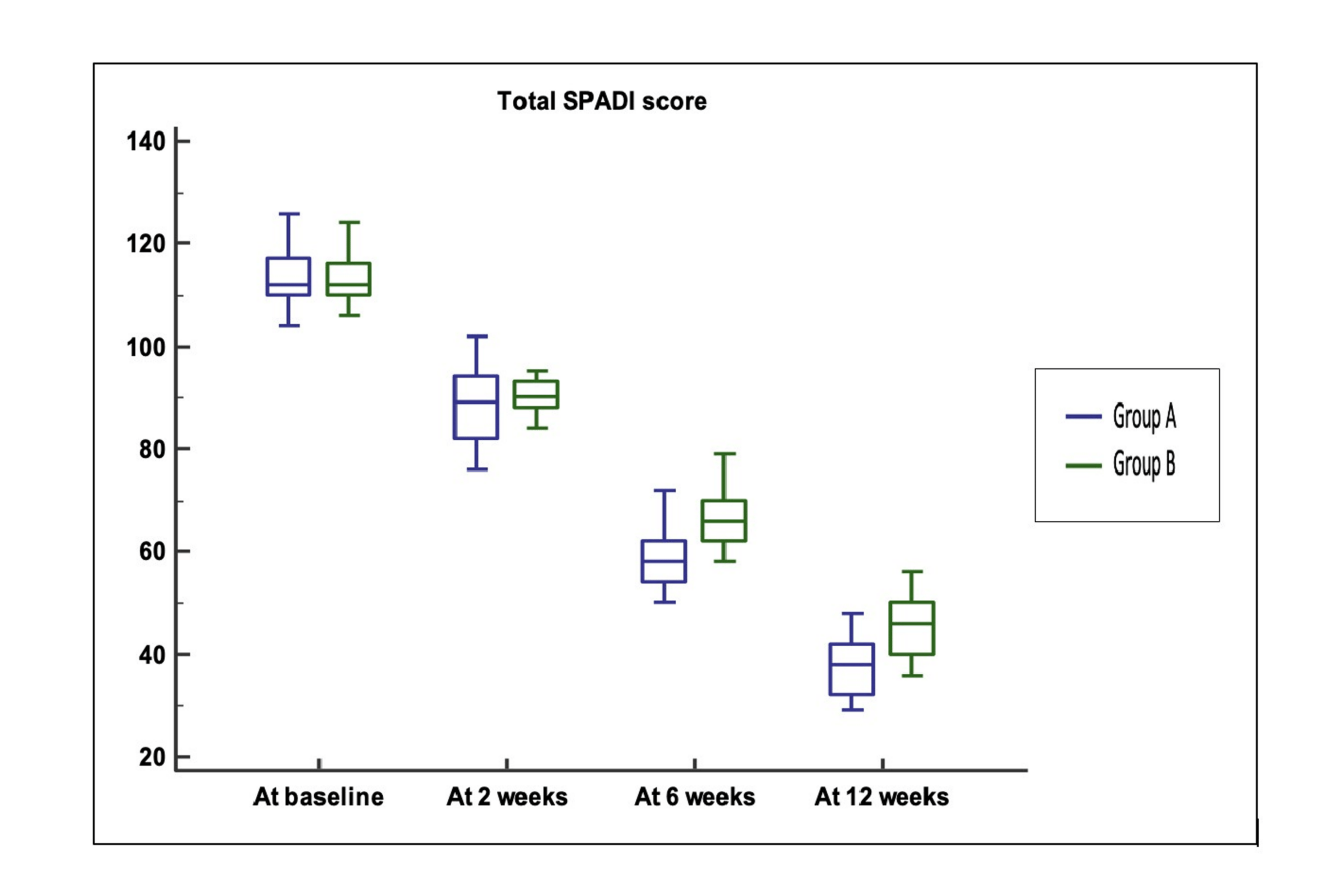
Total SPADI score comparison between Group A and Group B SPADI: Shoulder Pain and Disability Index The median (25th-75th percentile) total SPADI score at baseline was 112 (110–116.75) vs. 112 (110–116) (p-value = 0.947), and at two weeks, it was 89 (82.25–93.5) vs. 90 (88–92.75) (p-value = 0.498). The total SPADI scores at six and 12 weeks were significantly lower in Group A (58 (54.25–62) and 38 (32–42)) compared to Group B (66 (62.5–70) and 46 (40–50)) (p-values < 0.0001 for both).

Upon comparing Group A and Group B, no significant differences were observed in the total SPADI scores at baseline and at two weeks (Figure 4). The total SPADI scores at six and 12 weeks were significantly lower in Group A than in Group B (p-values < 0.0001).

**Figure 5:**
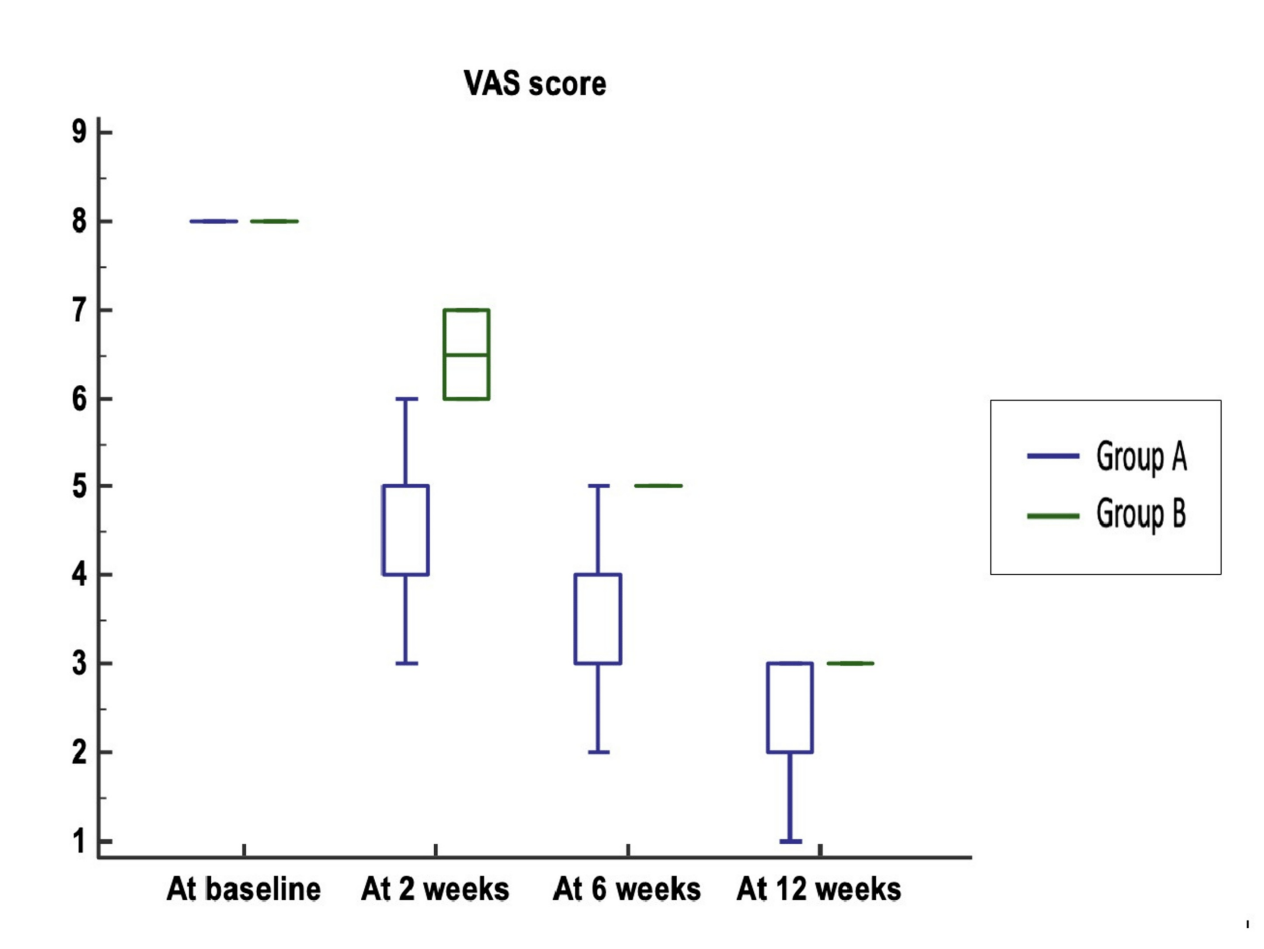
Comparison of VAS score between Group A and Group B VAS: Visual Analogue Scale The median (25th-75th percentile) VAS score was 8 (8–8) in both groups (p-value = 0.657). The VAS scores at two, six, and 12 weeks were significantly lower in Group A (5 (4–5), 4 (3–4), and 2 (2–3)) compared to Group B (6.5 (6–7), 5 (5–5), 3 (3–3)) (p-values < 0.0001 for all).

In the VAS scores, no significant difference was noted at baseline between Group A and Group B (Figure 5). VAS scores at two, six, and 12 weeks were significantly lower in Group A than in Group B (p-values < 0.0001).

**Figure 6:**
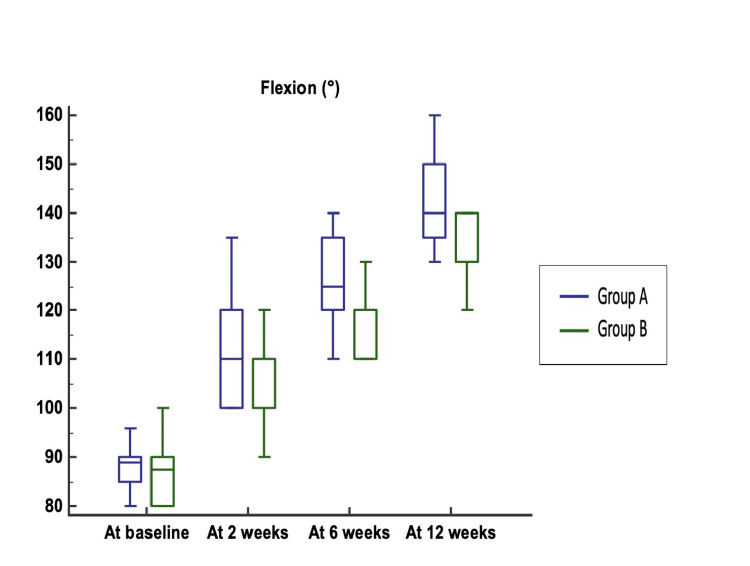
Comparison of flexion between Group A and Group B Median (25th-75th percentile) flexion was 89 (85–90)° vs. 87.5 (80–90)° (p-value = 0.487). Flexion at two, six, and 12 weeks was significantly higher in Group A (110 (100–120)°, 125 (120–135)°, 140 (135–150)°) compared to Group B (100 (100–110)°, 120 (110–120)°, 130 (130–137.5)°) (p-values = 0.011, 0.0009, and < 0.0001, respectively).

In ROM, no significant differences were perceived in flexion at baseline between Group A and Group B (Figure 6). Flexion at two, six, and 12 weeks was significantly higher in Group A than in Group B.

**Figure 7:**
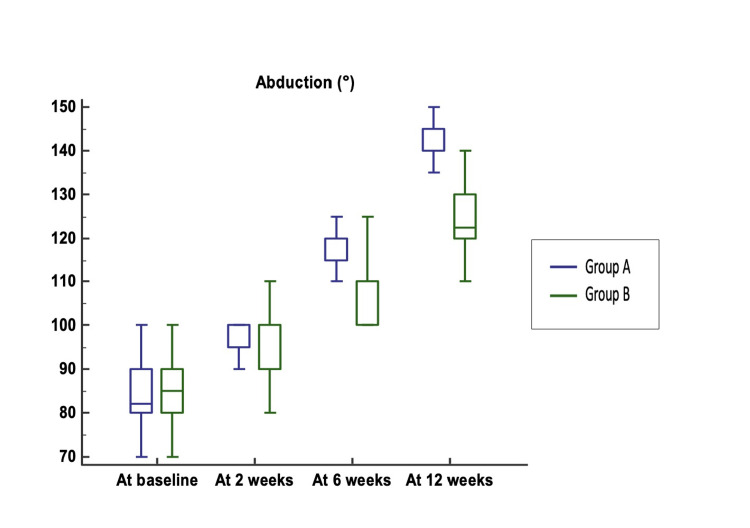
Comparison of abduction between Group A and Group B Median (25th-75th percentile) abduction was 82 (80–88.75)° vs. 85 (80–90)° (p-value = 0.128). Abduction (°) at two, six, and 12 weeks was significantly higher in Group A (100 (95–100)°, 120 (115–120)°, 140 (140–145)°) compared to Group B (90 (90–100)°, 110 (100–110)°, 122.5 (120–130)°) (p-values = 0.013, < 0.0001, and < 0.0001, respectively).

In abduction ROM, no significant difference was evident at baseline, but at two, six, and 12 weeks, it was significantly higher in Group A than in Group B (Figure 7).

**Figure 8:**
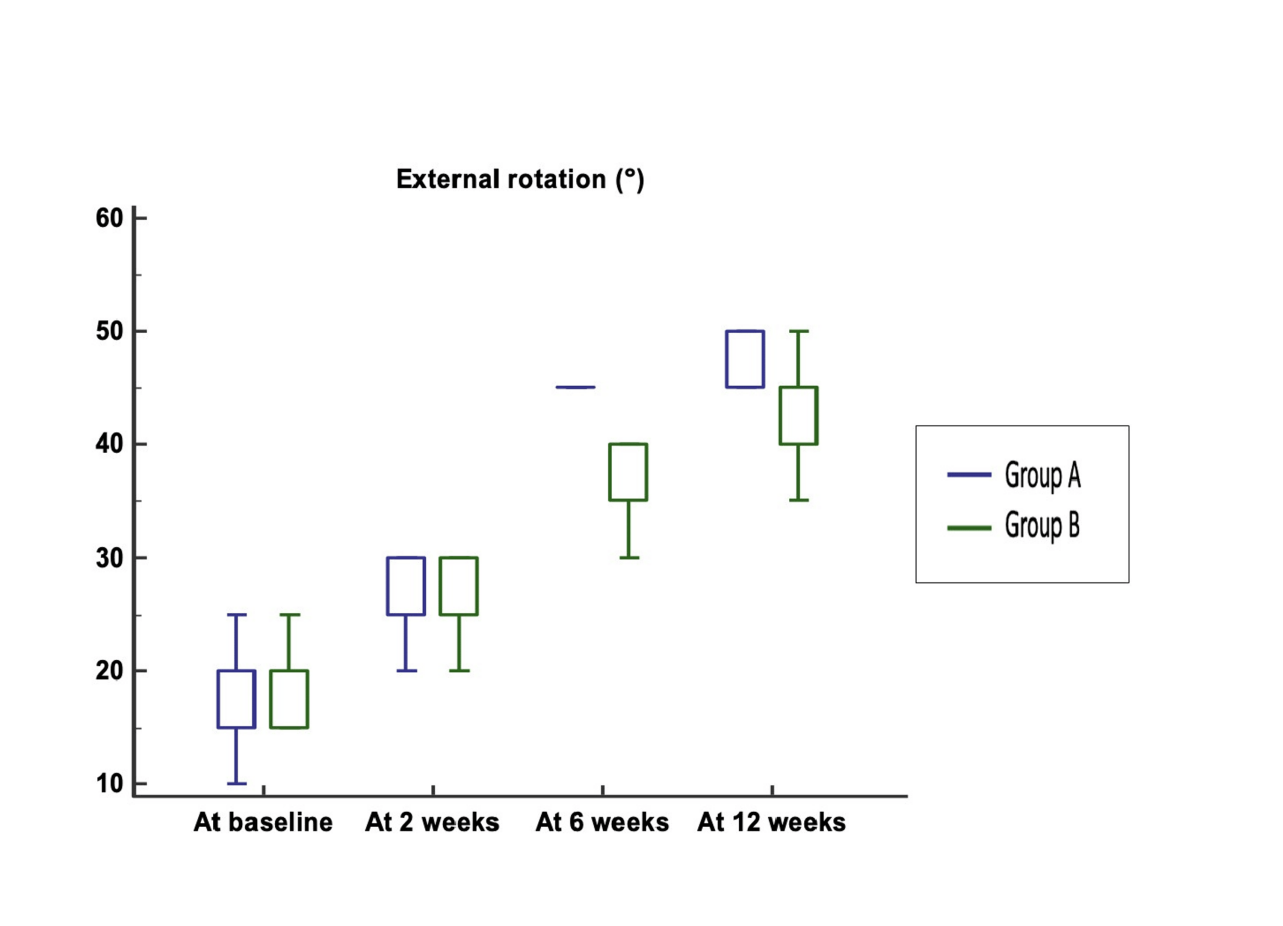
Comparison of external rotation between Group A and Group B Median (25th-75th percentile) external rotation at baseline was 15 (15–20)° vs. 20 (15–20)° (p-value = 0.213) and at two weeks was 25 (25–30)° in both groups (p value = 0.863). External rotation at six and 12 weeks was significantly higher in Group A (45 (45–45)° and 50 (45–50)°) compared to Group B (35 (35–40)° and 40 (40–45)°) (p-values < 0.0001 for both).

In external rotation, no significant differences were detected between Group A and Group B at baseline and at two weeks, but it became significantly increased in Group A compared with Group B at six and 12 weeks (Figure 8).

Tablet acetaminophen use was recorded in both groups and found to be insignificant.

## Discussion

PA shoulder is one of the commonly encountered conditions in outpatient musculoskeletal clinics, and its management depends on the patient’s presenting symptoms (only pain, only restricted ROM, or both). The condition is usually diagnosed based on patient history and detailed clinical examination (painful, stiff shoulder, and restricted ROM).

In recent years, regenerative medicine has introduced a promising new frontier in musculoskeletal pain management. PRP is used for the functional restoration of tissues with severe injuries or chronic diseases. PRP is a component of the blood that contains platelet-related growth factors above the normal level. It was proposed in 1997 as a low-cost, simple, safe, and minimally invasive product that could be directly and easily injected into injured tissues [9]. PRP contains various growth factors that aid in angiogenesis, vasculogenesis, cell growth, proliferation, and cartilage regeneration [10].

In this randomized controlled trial, all interventions were performed under USG guidance in both groups. This approach has been shown to enhance the precision of both intra-articular and nerve blocks and provide significant clinical improvement for the PA shoulder [11-13]. In addition, the use of USG facilitates accurate targeting and minimizes complications.

Demographically, PA shoulder often occurs in the fifth and sixth decades of life, and if it presents early, other systemic and local factors should be considered [6,14,15]. In this research, most patients were in the 5th decade of their life, which agrees with previous studies. A higher proportion of females (60%) was reported, which corroborates the findings from other investigations [15,16]. The right shoulder was more frequently affected than the left side (71.67% vs. 28.33%), corresponding to a study by Sonune et al. [17].

In this trial, both treatment groups-Group A (IA-PRP) and Group B (SSNB)-exhibited significant clinical improvement in all three parameters assessed (p-value < 0.0001) (total SPADI, VAS, and ROM) at two, six, and 12 weeks of follow-ups. Pain is a commonly evaluated parameter in most studies; the VAS scale is used to determine pain, and ROM is used to ascertain the improvement. These findings are consistent with multiple clinical trials supporting the individual efficacy of PRP [15,18,19] and SSNB [20-22] in the management of PA shoulder.

Nonetheless, on comparing both groups, crucial differences were revealed. No significant differences were observed at two weeks in total SPADI scores, suggesting comparable early benefits in terms of pain relief and function. However, by six and 12 weeks, Group A (IA-PRP) showed significantly lower scores than Group B (SSNB). This finding implies that overall function and quality of life increase more in the IA-PRP group than in the SSNB group in the medium term. Similarly, VAS scores were consistently and significantly lower in the PRP group, suggesting greater pain relief compared with the SSNB group.

Flexion and abduction ROM at two, six, and 12 weeks were significantly improved in the PRP group compared with the SSNB group. External rotation ROM was not significantly different at two weeks, whereas it improved significantly at follow-up over 6 and 12 weeks. These advancements are demonstrated in the PRP group’s consideration of the regenerative potential, as PRP may have effects on improving all phases of tissue repair, e.g., inflammatory, proliferative, and remodeling phases of capsular healing in PA [23].

In this trial, the medium-term superiority of parameters in the PRP group may be attributed to the sustained biological activities compared with SSNB. Bupivacaine used for SSNB has an immediate but transient analgesic effect; in contrast, PRP produces an enduring effect by modulating inflammatory and reparative processes. Hence, while SSNB offers early symptomatic relief, PRP provides a more long-lasting improvement in pain and function.

Importantly, in this study, all standard interventions were performed by a single expert physiatrist under USG guidance, ensuring fidelity and technical accuracy. All evaluations were done at regular intervals by another independent expert who was unaware of the procedure to minimize assessment bias. No complications were reported during the study period in patient populations, affirming the safety of both interventions.

Despite such promising findings, the relatively small sample size is one of the limiting factors affecting the universality of the outcomes. Furthermore, the follow-up period was limited to 12 weeks owing to time constraints. Given the chronic nature of PA shoulder, long-term follow-up studies are required to evaluate the sustainability of clinical benefits, particularly considering the fact that the regenerative effects of PRP may extend beyond the observed follow-up period.

## Conclusions

USG-guided IA-PRP and SSNB injections have demonstrated significant improvement in PA shoulder pain, each offering unique advantages. IA-PRP has shown greater pain relief and disability improvement in the short to medium term by promoting tissue healing and reducing inflammation. The choice of the therapeutic option may depend on patient preference, condition, and desired effect. From a futuristic perspective, a larger sample size and longer follow-ups are required to establish the comparative benefits of PRP over SSNB.
